# His-bundle Pacing as a Bailout Therapy for a Patient with Subclavian Stenosis and No Suitable Coronary Sinus Branch: A Double Whammy

**DOI:** 10.19102/icrm.2022.130902

**Published:** 2022-09-15

**Authors:** Wasim Rashid, Ibrahim Shah, Michael Soos, Khalil Kanjwal

**Affiliations:** ^1^Superspeciality Hospital, Government Medical College, Srinagar, India; ^2^McLaren Greater Lansing Hospital, Lansing, MI, USA

**Keywords:** Cardiac resynchronization therapy, His pacing, subclavian stenosis

## Abstract

We present an interesting case of an 88-year-old man who was referred to our arrhythmia service for an upgrade of his dual-chamber pacemaker to a biventricular pacemaker for right ventricular pacing–induced cardiomyopathy. The patient was found to have stenosis of the left subclavian vein. Here, we describe the approach used to perform venoplasty in this patient. After venoplasty of the left subclavian vein, the patient did not have suitable coronary venous anatomy for deployment of the coronary sinus lead. Subsequently, a His lead was implanted. We achieved significant narrowing of the QRS with good thresholds and other lead parameters. Through this case report, we seek to present our approach of venoplasty in patients with occluded venous access for either an upgrade or a de novo implant.

## Case presentation

**Figure 1:**
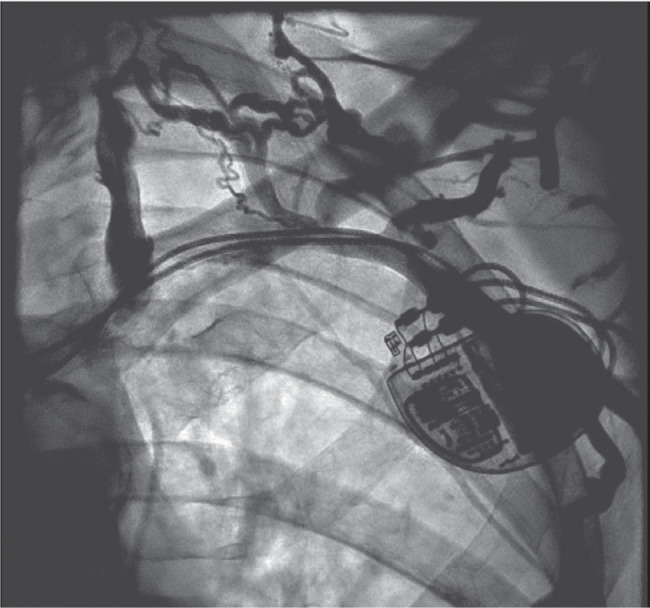
A venogram of the left subclavian venous system revealed subclavian vein stenosis and collaterals.

**Figure 2:**
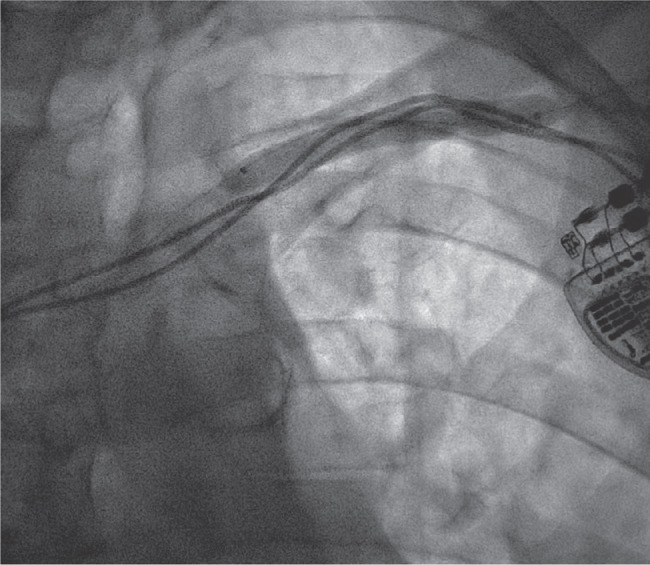
A 0.035-in angled Navicross catheter was advanced over an angled glidewire advantage and was successful in crossing the total occlusion.

An 88-year-old man with a history of complete heart block status after dual-chamber pacemaker implantation was sent to our arrhythmia clinic for the management of right ventricular (RV) pacing–induced cardiomyopathy. He had a history of paroxysmal atrial fibrillation and chronic kidney disease (CKD) with a creatinine level of 1.5 mg/dL. He was also suffering from chronic lymphocytic leukemia and had a chemotherapy port in the right subclavian vein. The patient was experiencing heart failure (HF) symptoms, including shortness of breath and leg swelling. His echocardiogram showed an ejection fraction (EF) of 40%. A stress test was performed, which did not reveal any ischemia. Given his need for RV pacing and cardiomyopathy, an upgrade of his dual-chamber pacemaker to a biventricular (BiV) pacemaker was offered. The patient was brought to the electrophysiology lab in the post-absorptive state. Routine venography of the left subclavian venous system was performed, which revealed subclavian vein stenosis **(Figure 1)**, thus precluding the implantation of the coronary sinus (CS) lead. The options included implanting a new system from the right side, except that the patient had a chemotherapy port on that side, or a surgical left ventricular (LV) lead implantation. Ultimately, we decided to perform a balloon venoplasty of the left subclavian vein stenosis. A left antecubital vein access was upsized to a 6-French Terumo slender sheath (Terumo Medical Corporation, Somerset, NJ, USA). A repeat venogram demonstrated the occlusion of the left subclavian vein and collaterals from the left cephalic as well as the basilic vein into the left internal jugular vein. At the site of previous leads, there was no flow noted **(Video 1)**. A 0.035-in angled Navicross catheter was advanced over an angled glidewire advantage, which, by gentle manipulation, was successful in crossing the total occlusion **(Figure 2)**. The tip of the catheter was then placed in the inferior vena cava, and the angled glidewire advantage was exchanged with a Wholey wire. An 8.0- × 6-mm balloon was advanced; however, the balloon failed to cross the obstruction. The Wholey wire was exchanged with a 0.014 balance middleweight 300-cm wire. A 4.0- × 80-mm ultraverse balloon **(Figure 2)** was used for balloon angioplasty, which was upsized to an 8.0- × 60-mm balloon. Balloon angioplasty was performed with an 8.0-mm balloon at 6 atm **(Video 2)**. Repeat cineangiography revealed adequate flow across the previously blocked subclavian vein without any dissection or perforation.

**Figure 3:**
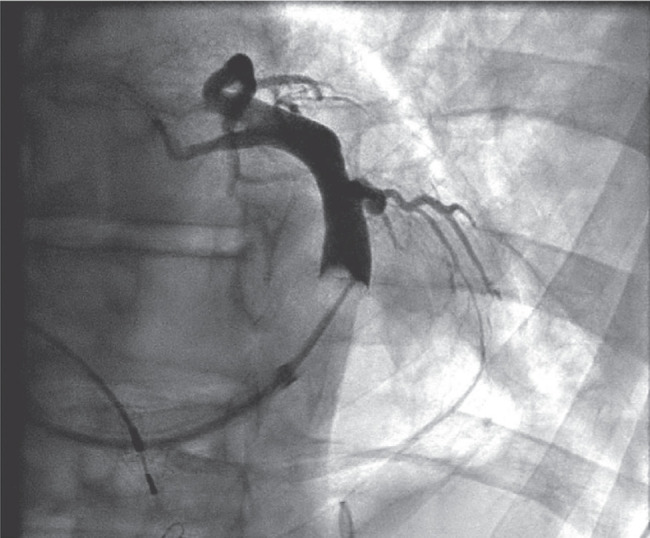
Coronary sinus venography was performed, which revealed a tortuous lateral branch that tapered abruptly.

**Figure 4:**
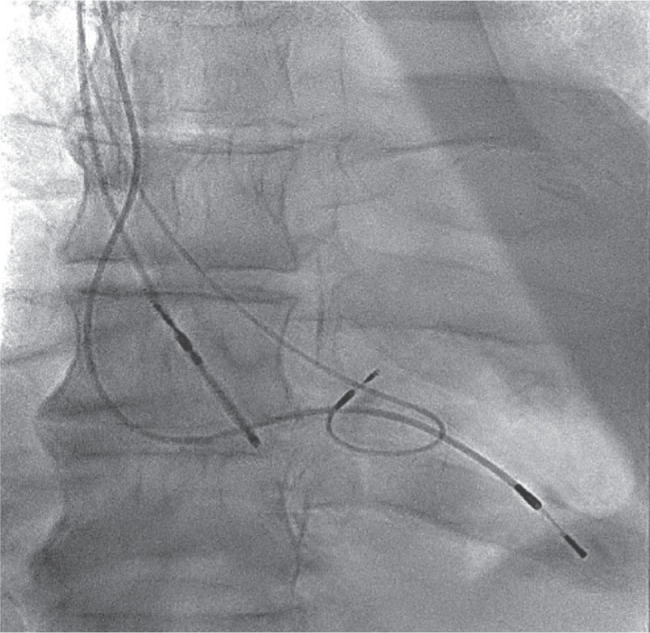
The final position of the His lead.

**Figure 5:**
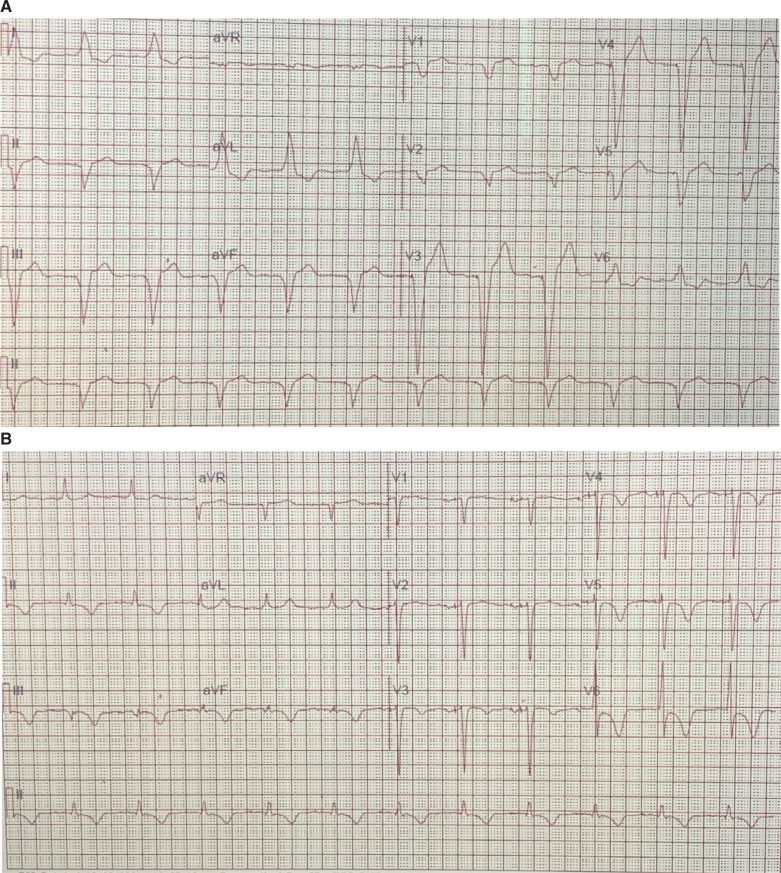
Pre- **(A)** and postprocedure **(B)** electrocardiograms showed a significant narrowing after His lead implantation.

Subsequently, the pocket was opened, and the device and the leads were dissected out. Using a micropuncture needle set, subclavian vein access was obtained. A Worley delivery sheath was advanced over the guidewire. CS cannulation was performed without any difficulty. A CS venogram was recorded, which revealed a tortuous lateral branch that tapered abruptly **(Figure 3)**. Multiple attempts at placing the CS lead over the angioplasty guidewire failed. A renal vein subselector was used; however, we could not cannulate the side branch with a guidewire. The options were to perform an angioplasty of the CS lead or conduction system pacing (CSP) either with His-bundle pacing (HBP) or left bundle pacing. Given his history of CKD and earlier subclavian venoplasty (SV), it was decided to limit further use of the contrast and instead perform HBP. A Medtronic SelectSecure 3830 lead (Medtronic Inc., Minneapolis, MN, USA) was advanced over the C315 delivery system. Pace-mapping was performed in the area where we had a good His signal. Selective and non-selective capture of the His bundle was demonstrated. The helix was delivered by clockwise rotation of the lead **(Figure 4)**. The pacing threshold was 1.2 V at 0.4 ms. An electrocardiogram showed a significant narrowing compared to baseline **(Figures 5A and 5B)**.

The patient was subsequently discharged home after an overnight observation. He was followed up in the clinic at 6 weeks and was feeling much better with improvements in his symptoms of fluid retention and shortness of breath. His EF on follow-up was noted to be 50%.

## Discussion

This case illustrates the anatomical barriers that can be encountered during a device upgrade of a patient with multiple comorbidities and the tools and techniques available to an implanting physician in the current era to overcome and troubleshoot these problems. The decision to upgrade the pacemaker to a BiV pacemaker was made in view of the symptomatic HF with LV dysfunction, a high percentage of RV pacing, and the absence of inducible ischemia.

The first anatomical obstacle in our case was the subclavian occlusion precluding access to implant an LV lead. The incidence of subclavian vein stenosis after device implantation varies widely in the literature, ranging from 30%–50%.^1,2^ Previous use of transvenous temporary leads, LVEF < 40%, and advanced age (>65 years) were found to be independent risk factors for a higher incidence of venous occlusion. Most of the patients in these studies were asymptomatic due to the collateral venous circulation that developed as the stenosis progressed.

In our situation, one of the options was to implant a cardiac resynchronization therapy (CRT) pacemaker device from the other side. However, the patient had a chemotherapy port on the right side, making that route of access unavailable. Crossing intraluminal occlusions followed by SV has been demonstrated to be a safe alternative. The largest series reporting the use of SV for lead implantation included 373 cases documented over an 11-year period. Successful access was achieved in 371 of 373 cases with no adverse clinical outcomes, including distal embolization, venous disruption, and damage to the leads.^3^ The 2017 Heart Rhythm Society expert consensus statement includes SV as an option when venous access becomes an issue due to occlusion of the desired access point.^4,5^ Once the access was established in our case with SV, another anatomical challenge emerged in an attempt to implant an LV lead.

We know that CRT is the only known non-pharmacologic HF therapy that improves cardiac function, functional capacity, and survival while decreasing cardiac workload and hospitalization rates in chronic systolic HF and conduction system disease.^6,7^ However, CRT is not without its limitations. One major limitation of BiV pacing in CRT is the failure of LV lead deployment due to limitations in CS anatomy,^8^ and the standard of care suggests epicardial LV lead insertion as the next alternative. Historically, in the Multicenter Automatic Defibrillator Implantation Trial–Cardiac Resynchronization Therapy (MADIT-CRT), 7.5% of patients (n = 82) who were assigned to the CRT-implantable cardioverter-defibrillator (ICD) group received an ICD-only device during the trial because of technical difficulties in positioning the LV pacing lead in the coronary vein.^9^ Overall, an unfavorable CS anatomy precludes the delivery of an LV lead placement in 5%–8% of patients, and the lead position is suboptimal in another 15%–20% of patients.^10^ In a contemporary meta-analysis of 29,503 patients, the overall rate of failure of implantation of an LV lead was 3.6% (95% confidence interval [CI], 3.1%–4.3%). The rate of failure in studies that commenced before 2005 was 5.4% (95% CI, 4.4%–6.5%), and that in studies performed from 2005 onward was 2.4% (95% CI, 1.9%–3.1%; *P* < .001). The causes of failure (reported for 39% of failures) also changed over time. Failure to cannulate and navigate the CS decreased from 53% to 30% (*P* = .01), and the absence of any suitable, acceptable vein increased from 39% to 64% (*P* = .007).^8^ Our case was representative of this trend, with no suitable branch for LV lead placement found after CS cannulation. Therefore, we had to seek alternatives to an LV lead in our case.

Presently, CSP serves as an attractive bailout strategy in patients with a lack of coronary venous access, diaphragmatic pacing, and/or failure to respond to classic CRT.^7^ The 2021 European Society of Cardiology guidelines^11^ recommend HBP as a treatment option together with other techniques such as surgical epicardial lead (class IIa level of evidence B indication) in CRT candidates in whom CS lead implantation is unsuccessful.

HBP was first reported in humans in 2000^12^ and is steadily gaining interest for providing a more physiological alternative to RV pacing. It may also correct intraventricular conduction delay in a subset of patients, thereby providing an alternative to BiV pacing for treating HF. There is growing evidence, mainly from observational studies, that HBP may be safe and effective in these settings, although large randomized controlled trials (RCTs) and long-term follow-up data are still lacking.^13^ Lustgarten et al. were the first to design a prospective crossover study in which patients received both HBP and traditional CS LV leads. They found comparable results at 1 year of follow-up between functional outcomes (eg, New York Heart Association [NYHA] functional class, 6-min walk test, and quality-of-life [QoL] assessment) and echocardiographic outcomes (eg, LVEF, LV end-diastolic and end-systolic volumes, mitral regurgitation jet area, and LV outflow tract flow velocity integral). The study was an important proof of concept for HBP-CRT as a potential first-line strategy for CRT.^14^ In another study, Vijayaraman et al. showed successful HBP in 90.6% of patients (n = 32), 14 of whom had failed CS leads and 2 who were non-responders to BiV-CRT. They observed QRS narrowing and improvements in LVEF and NYHA functional class, reiterating that HBP in lieu of an LV lead is also feasible.^15^ Ajijola et al. evaluated 21 patients with an indication for CRT implant to incorporate a His-bundle lead for CRT in lieu of a CS lead. HBP implantation was successful in 76% of patients (16/21 patients) with significant narrowing of the QRS duration and improvement in NYHA class, LVEF, and LV internal dimensions in diastole at 6 months of follow-up.^16^ Sharma et al. assessed 106 patients with CRT indications for HBP as a rescue strategy for failed LV lead or non-response to BiV pacing, or as a primary strategy for atrioventricular (AV) block, bundle branch block, or high ventricular pacing burden as an alternative to BiV pacing. The study reported a 90% success rate with significant QRS narrowing, an increase in LVEF, and an improvement in NYHA class after a mean follow-up of 14 months.^17^

The first prospective RCT to compare corrective HBP versus BiV pacing for CRT was the His Bundle Pacing vs. Coronary Sinus Pacing for Cardiac Resynchronization Therapy (His-SYNC) pilot trial, which was an investigator-initiated study conducted at 7 centers in the United States.^18^ In this first randomized pilot trial, His-CRT did not demonstrate significant improvements in electrocardiographic or echocardiographic parameters compared to BiV-CRT. This study was underpowered to detect differences of <10% between groups, and the existence of a type II error cannot be excluded. Importantly, an intention-to-treat analysis in the presence of high crossover rates cannot directly assess treatment efficacy.

Challenges with HBP include the complexity of implant and significant learning curve, higher acute pacing thresholds with a rise in follow-up, and diminutive R-wave sensing. What is sorely needed is both refinement in delivery tools of His leads and more data on clinical outcomes after HBP in patients with HF. The His Optimised Pacing Evaluated for Heart Failure Trial (HOPE-HF) (NCT02671903) will provide some data in this regard, as it evaluates the use of HBP in HF patients with long AV delay.

We were able to achieve an acceptable threshold for the His lead and QRS narrowing, which is a surrogate for electrical resynchronization. The patient was seen for follow-up at 6 weeks and 3 months. He reported an improvement in his symptoms, and an echocardiogram revealed an EF of 50% at 3 months.

## Conclusion

The cardiac device–implanting physician has multiple tools and techniques available in their armamentarium to overcome some of the anatomical challenges during device implants, especially during device upgrades. One must be familiar with these contemporary tools and techniques in order to deliver the best results to the patient in terms of procedural success as well as long-term clinical outcomes.

## Figures and Tables

**Video 1:** Venography demonstrated occlusion of the left subclavian vein and collaterals from the left cephalic as well as the basilic vein into the left internal jugular vein. At the site of previous leads, no flow was seen.

**Video 2:** Balloon angioplasty was performed with an 8.0-mm balloon at 6 atmospheric pressures.
